# *Ceropegia gengmaensis* (Apocynaceae), a new species from Yunnan, China

**DOI:** 10.3897/phytokeys.276.197333

**Published:** 2026-06-15

**Authors:** Peng-Rui Luo, Ang Liu, Rui Tong, Xin-Jian Zhang, Zi-Yi Wang, Tao Deng, David J. Goyder, Hang Sun

**Affiliations:** 1 State Key Laboratory of Plant Diversity and Specialty Crops, Kunming Institute of Botany, Chinese Academy of Sciences, Kunming 650201, Yunnan, China Royal Botanic Gardens, Kew London United Kingdom; 2 College of Life Sciences, University of Chinese Academy of Sciences, Beijing 100049, China State Key Laboratory of Plant Diversity and Specialty Crops, Kunming Institute of Botany, Chinese Academy of Sciences Kunming China; 3 Hunan Botanical Garden, Changsha 410116, Hunan, China College of Life Sciences, University of Chinese Academy of Sciences Beijing China; 4 Royal Botanic Gardens, Kew, TW9 3AE London, UK Hunan Botanical Garden Changsha China

**Keywords:** Ceropegieae, Chloroplast, Karst Landform, Morphology, Southwest China

## Abstract

*Ceropegia gengmaensis* P.R.Luo, A.Liu & H.Sun, a new species from Gengma County, Yunnan, Southwest China, is described. Both morphological characteristics and chloroplast phylogenetic analysis strongly support its placement within *C.* sect. *Chionopegia* H.Huber. Molecular data further reveal that *C. gengmaensis* is closely related to *C. salicifolia*, *C. mairei*, and *C. dolichophylla*, yet it can be distinguished from them readily by its stem indumentum, leaf shape, and floral morphology, particularly the shape of the corolla tube, and features of the lobes. A detailed line drawing of this new species is also provided.

## Introduction

Traditionally, the genus *Ceropegia* L. was considered to comprise about 240 species worldwide (Huber 1957; Li et al. 1995; Meve and Liede-Schumann 2007; Kambale and Yadav 2015; Endress et al. 2018; Gilbert 2020). However, molecular phylogenetic studies have demonstrated that this circumscription does not represent a monophyletic group (Bruyns et al. 2015; Bruyns et al. 2017). To achieve monophyly and ensure nomenclatural stability, Bruyns proposed a broadened circumscription that incorporates the traditional *Brachystelma* R.Br. as well as all stapeliad genera, thereby making *Ceropegia* the largest genus of Apocynaceae Juss., comprising 63 sections and more than 700 species (Bruyns et al. 2017). Under this expanded concept, World Flora Online currently recognizes 863 species, including 424 species traditionally treated as *Ceropegia* and *Brachystelma* (World Flora Online 2026).

Recent taxonomic studies on *Ceropegia* in China have highlighted the influence of the region’s complex geological evolution and climatic diversity on species diversification and distribution patterns within a broader Eurasian context (Chen et al. 2018). In these works, five new species were described, *Ceropegia paohsingensis* Tsiang & P.T. Li was treated as a synonym of *Ceropegia driophila* C. K. Schneider, and the *Ceropegia angustilimba* Merr., previously reduced to synonymy, was reinstated as distinct from *Ceropegia trichantha* Hemsl. As a result, the total number of currently recognized species of *Ceropegia* in China now stands at 26. This includes 22 species of *C.* sect. *Chionopegia* and 4 of *C.* sect. *Tiloris*, with two species formerly placed in *Brachystelma* now incorporated (Wu et al. 2019; Ma et al. 2022a, 2022b; Luo et al. 2023; Xie et al. 2023; Luo et al. 2024; Luo et al. 2026).

During a recent floristic investigation in Gengma County, Yunnan Province, southwest China, an unknown species of *Ceropegia* was collected. It belongs to *Ceropegia* sect. *Chionopegia* H.Huber, as it has no tuber but fleshy roots, a corolla with a slender tube globose at the base, and lobes coherent at the apex (Bruyns et al. 2017). *Ceropegia gengmaensis* is closely related to *Ceropegia salicifolia* H. Huber, *Ceropegia mairei* (H.Lév.) H.Huber and *Ceropegia dolichophylla* Schltr., yet it is readily distinguishable from all three by its distinct vegetative and floral morphology. Therefore, we describe *C. gengmaensis* here as a new species.

## Materials and methods

### Morphological observations

**Figure 1.**
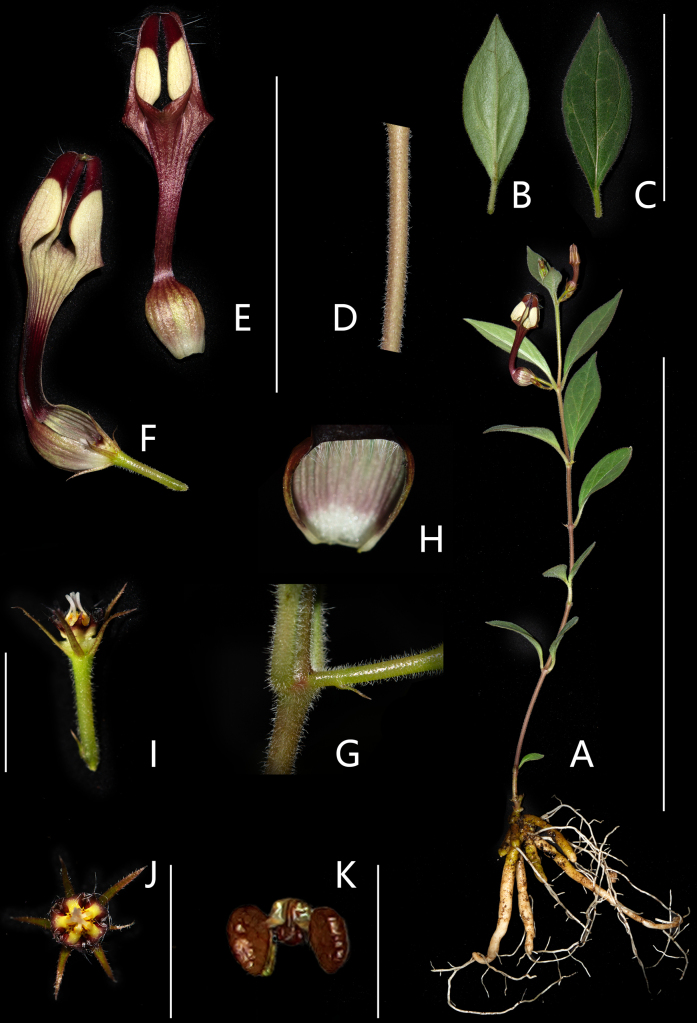
Morphological features of *Ceropegia gengmaensis* based on living plants collected from the type locality. **A**. Plant showing the rootstock; **B, C**. Leaf: B, abaxial surface and C, adaxial surface; **D**. Stem; **E, F**. Corolla: E, corolla from the outside with its base cut off and F, corolla with the tube dissected longitudinally; **G**. Small peduncle, part of the pedicel and petiole; **H**. White hairs on the inside of the base of the tube; **I**. Side view of gynostegium; **J**. Gynostegium from above; **K**. Pollinarium. Scale bars: 15 cm (**A**); 5 cm (**B, C**); 4 cm (**D, E, F**); 1 cm (**G, H, I**); 0.8 cm (**J**); 0.5 mm (**K**).

**Figure 2.**
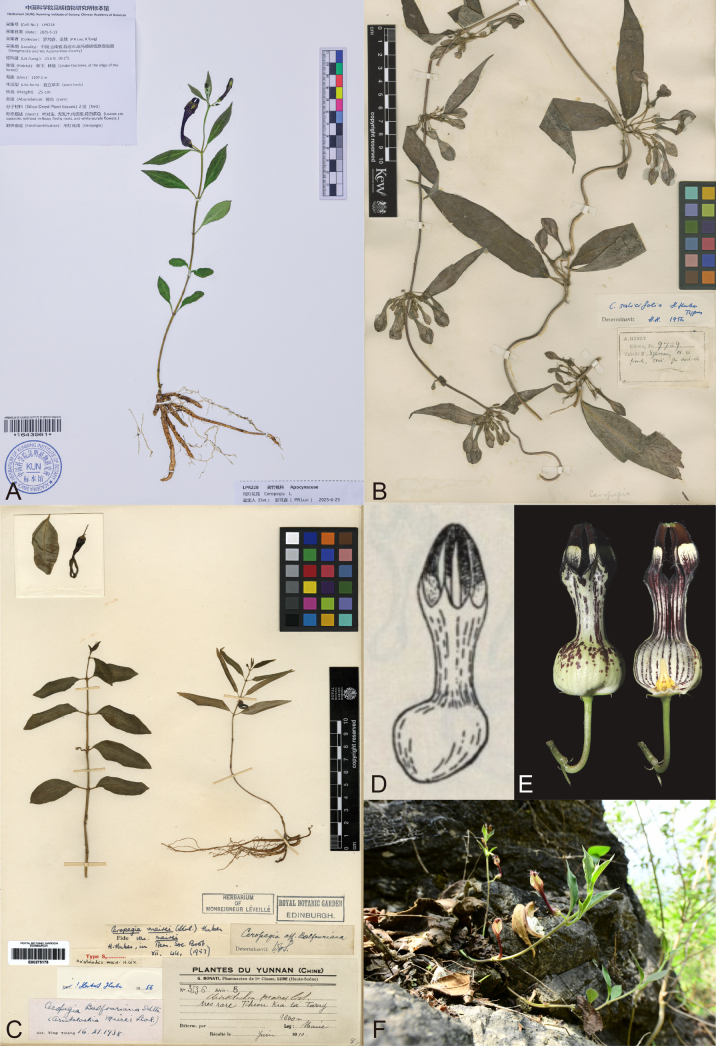
**A**. Holotype of *Ceropegia gengmaensis* (*P. R. Luo 228*, KUN1643961!); **B**. Holotype of *C. salicifolia* (*Henry, A. 9729*, K000857835!); **C**. Specimen *E. E. Maire 3536B*, (E00275175!) cited as “Typus” by H. Huber (1957); **D**. Sketch of *C. mairei* in H. Huber, Mem. Soc. Brot. 12: 43. 1957.; **E**. Flowers characteristics of *C. mairei*, photographed from living plants at the collection locality of Zhang T. 16CS12570, KUN1455767!; **F**. Plant of *C. gengmaensis* in habitat.

Morphological assessments were performed on both fresh material and herbarium specimens, employing stereomicroscopy, a high-resolution extended depth-of-field imaging system, and precision vernier calipers. To ensure rigorous taxonomic identification, a comprehensive literature survey was undertaken, and specimens housed in KUN and PE were examined directly, whereas collections from other herbaria were reviewed online via the Chinese Virtual Herbarium (**CVH**, http://www.cvh.ac.cn). Protologue information was obtained from the Biodiversity Heritage Library (**BHL**, https://www.biodiversitylibrary.org), and specimen records were cross-validated through CVH and JSTOR Global Plants (https://jstor.botanicalgarden.cn). Diagnostic morphological traits differentiating the new species from *C. salicifolia*, *C. mairei* and *C. dolichophylla* are illustrated and summarized in Figs 1, 2, 4 and Table 1.

**Table 1.** Diagnostic characters of *Ceropegia gengmaensis* and comparison with other related species.

Characters	*C. gengmaensis*	*C. salicifolia*	*C. mairei*	*C. dolichophylla*
Stem	Erect to sprawling, up to 300 mm long, uniformly finely puberulent	Twining to 150 mm, glabrous	Erect or twining, to 350 mm, minutely puberulent	Twining, to 150 mm long, glabrous
Leaf shape	Elliptic	Elliptic	Elliptic to elliptic-lanceolate	Linear-lanceolate
Leaf size	20–55 × 10–25 mm	60–150 × 10–21 mm	10–50 × 4–23 mm	50–120 × 5–20 mm
Peduncle	Subsessile	0–10 mm long, when present pubescent along 2 lines	0–20 mm long	2–30 mm long, sparsely pubescent
Pedicel	5–10 mm long, ca. 1 mm in diameter, finely pilose	10–15 mm long, glabrous	4–17 mm, sparsely puberulent	10–15 mm long, glabrous
Corolla length	30–50 mm	30–36 mm	43–49 mm	22–47 mm
Corolla shape and color	Basal swelling ovoid, gradually narrowed and increasing from the middle of tube gradually to 5–10 mm	Inflated base narrowing and widening at throat	Strongly inflated in basal 1/3–2/3	Inflated at base, narrowing and then widening at base of lobes
Width of ovoid cage formed by lobes	1.5 times as wide as basal inflation	2–3 times as wide as basal inflation	0.8–1 times as wide as basal inflation	1.5 times as wide as basal inflation
Corolla lobes	ligulate, red outside, white inside with reddish-purple apices, margins strongly reflexed, exposing.	Triangular, notched at apex, pale reddish-brown, margins slightly recurved	Linear-oblong, strongly revolute, with apex strongly incurved inward	Triangular, pale, with a slender and darker apical part; coherent at the apex; keeled; pilose on the inner surface; sometimes bearing conspicuous white or purple marginal vibratile hairs.

### Molecular analyses

To clarify the phylogenetic placement of *Ceropegia gengmaensis* sp. nov., we reconstructed phylogenetic relationships using complete chloroplast genomic data. In total, eleven *Ceropegia* plastomes were newly sequenced and assembled in this study. Additional chloroplast genome sequences from related species of *Ceropegia*, together with *C. nilotica* as an outgroup, were retrieved from GenBank (Table 2) (Wang et al. 2023).

**Table 2.** Species and accession numbers used in the phylogenetic analysis.

Name in Fig. 3	Voucher	Locality for voucher	GenBank accessions
*Ceropegia sunhangiana*1	P.R.Lu*o sx0*01	Qiaojia, Yunnan, China	OR260539
*Ceropegia sunhangiana*2	Z.X.Ren *12CS504*9	Qiaojia, Yunnan, China	/
*Ceropegia semifusca*	J.D.Ya *24CS2594*8	Kunming, Yunnan, China	/
*Ceropegia sinoerecta*	P.R.Luo *72*6	Binchuan, Yunnan, China	/
*Ceropegia salicifolia*1	Z.Y.Wang *280*	Puer, Yunnan, China	/
*Ceropegia salicifolia*2	Z.X.Ren *2012-RZX-0011*	Puer, Yunnan, China	/
*Ceropegia gengmaensis*1	P.R.Luo *22*8	Gengma, Yunnan, China	/
*Ceropegia gengmaensis*2	A.Liu LAYNGM 01	Gengma, Yunnan, China	/
*Ceropegia dolichophylla*2	C.F.Zhang *6482*	Kunming, Yunnan, China	OP133586
*Ceropegia dolichophylla*3	C.F.Zhang *DY06*2	Guiyang, Guizhou, China	OP133580
*Ceropegia dolichophylla*1	R.Tong *TR250723*	Kunming, Yunnan, China	/
*Ceropegia dolichophylla*4	R.Tong *TR25060*1	Guiyang, Guizhou, China	/
*Ceropegia pubescens*	P.R.Luo *QTP-Sun74*1	Nielamu, Xizang, China	/
*Ceropegia aridicola*	F.M.Yang	Lijiang, Yunnan, China	/
*Ceropegia jinshaensis*	J.D.Ya *15CS1121*3	Lijiang, Yunnan, China	/
*Ceropegia mairei*	Zhang T. *16CS12570*	Panzhihua, Sichuan, China	/
*Ceropegia nilotica*	C.F.Zhang *6230*	*/*	OP133584

Following the sectional framework proposed by Bruyns et al. (2017), the final dataset encompassed 16 representative species from three closely allied sections—*C.* sect. *Chionopegia* , *C.* sect. *Callopegia*, and *C.* sect. *Tiloris*. Sequence alignment was performed in MAFFT v7.490 using default settings (Katoh and Standley 2013), and poorly aligned regions were pruned with trimAl v1.4 (Capella-Gutiérrez et al. 2009). The aligned sequences dataset has been deposited in figshare (Luo et al. 2026; https://doi.org/10.6084/m9.figshare.32006028).

**Figure 3.**
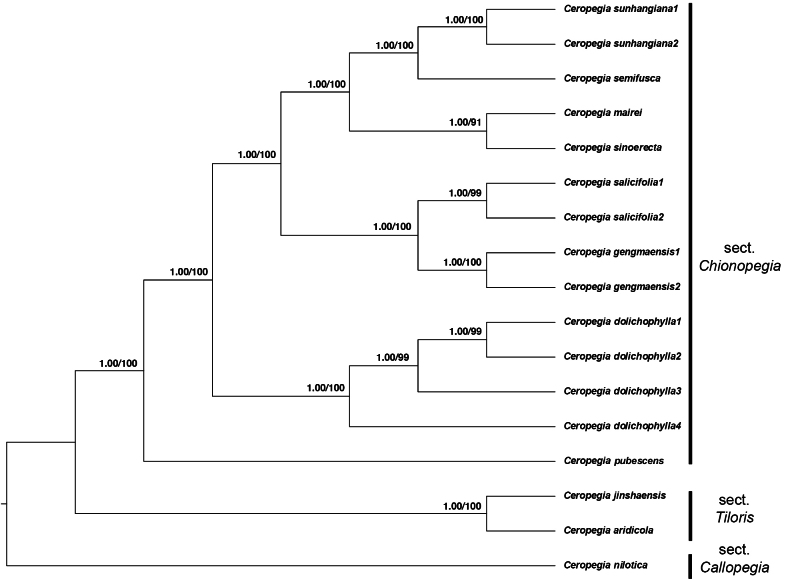
Phylogenetic tree for part of *Ceropegia* based on the complete chloroplast sequences. Support values for the branches are bootstrap support (BS) from ML, Bayesian posterior probability (PP) from BI.

**Figure 4.**
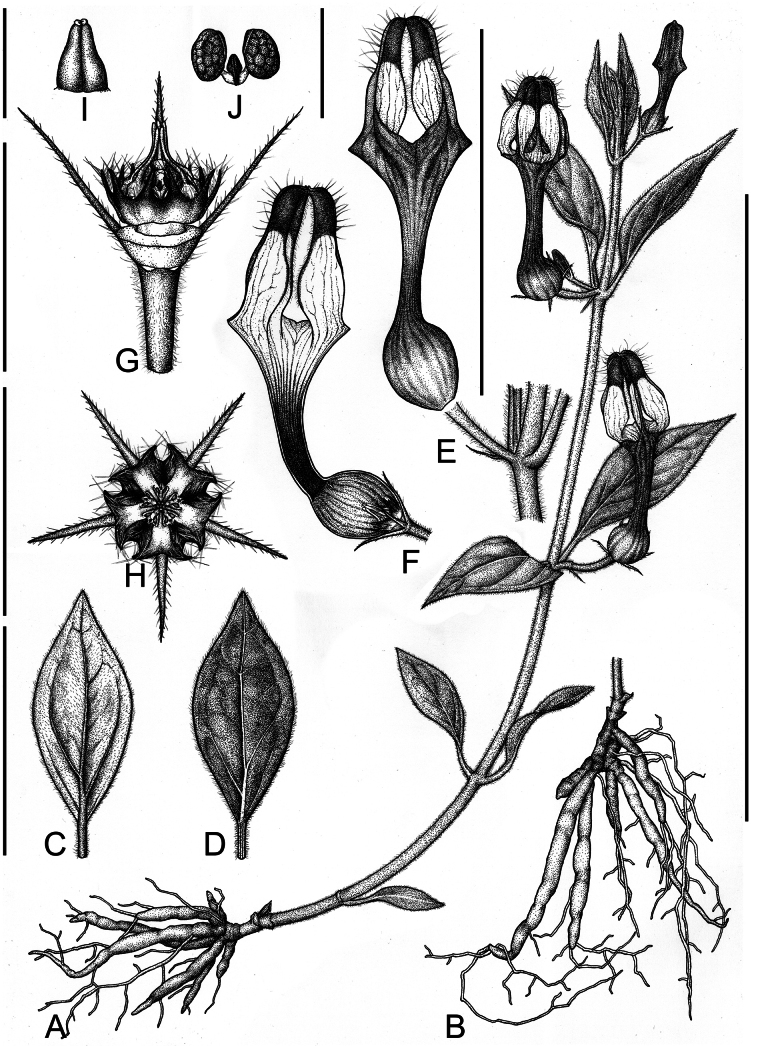
*Ceropegia gengmaensis*. **A, B**. Plant showing the rootstock; **C, D**. Upper and lower sides of leaf; **E**. Inflorescence with bases of adjoining leaves; **F**. Corolla tube dissected longitudinally; **G**. Side view of gynostegium (with sepals and pedicel shown); **H**. Top view of gynostegium with sepals shown; **I**. Ovary; **J** Pollinarium. Scale bars: 15 cm (**A, B**); 5 cm (**C, D**); 4 cm (**E, F**); 1 cm (**G**); 0.8 cm (**H**); 0.3 cm (**I**); 0.5 mm (**J**).

Phylogenetic reconstruction was carried out using both maximum likelihood (ML) and Bayesian inference (BI) approaches. ML analysis was implemented in RAxML-NG v1.2.2 under the GTR+G substitution model (Kozlov et al. 2019), whereas BI was performed in MrBayes as integrated in Geneious using the HKY85+G model (Kearse et al. 2012; Ronquist et al. 2012). The Markov Chain Monte Carlo (MCMC) run consisted of 1,100,000 generations with four chains (heated chain temperature = 0.2), sampling every 200 generations, and the first 100,000 generations were discarded as burn-in. *Ceropegia nilotica* (GenBank accession OP133584) was designated as the outgroup, and a random seed ensured reproducibility.

Branch support was assessed using Bayesian posterior probabilities (PP) and ML bootstrap percentages (BS). Nodes with PP ≥ 0.95 and BS ≥ 75% were regarded as strongly supported.

## Results

### Morphology and taxonomy

Compared with other *Ceropegia* species in China, *C. gengmaensis* is morphologically distinctive, yet shows affinities with *C. salicifolia*, *C. mairei* and *C. dolichophylla*, all members of *C.* sect. *Chionopegia* (as corroborated by our molecular results). However, it can be readily distinguished from these congeners by a combination of vegetative and floral characters. *C. gengmaensis* differs from *C. salicifolia* in being an erect to sprawling herb rather than a twining vine. Its leaves are elliptic and relatively small (2.0–5.5 × 1.0–2.5 cm), whereas those of *C. salicifolia* are lanceolate and substantially longer (6–15 × 1–2.1 cm). The stems of *C. gengmaensis* are uniformly finely puberulent and usually bear solitary flowers next to the axils, whereas *C. salicifolia* has glabrous stems and produces cymose inflorescences with 13–20 flowers. Floral morphology further distinguishes the two species: the corolla of *C. salicifolia* is markedly larger, with the upper inflated portion of the tube nearly twice as wide as the basal region, whereas *C. gengmaensis* exhibits a slenderer corolla with narrower lobes that are only slightly expanded above the throat.

In addition, *C. gengmaensis* can be clearly distinguished from *C. mairei* by several key floral characters. In *C. mairei*, the corolla tube is strongly inflated at the base and bears a dense zone of long white hairs on the inner surface, particularly around the upper part of the inflated portion near its mouth. In contrast, *C. gengmaensis* has a much less inflated basal tube, and the indumentum inside the corolla is restricted to a distinct annular ring of white hairs rather than being distributed throughout the inner surface.

*C. dolichophylla* is a twining or climbing species with glabrous stems reaching up to 1.5 m in length, and bears much longer, linear–lanceolate leaves. By comparison, *C. gengmaensis* is an erect to sprawling herb with densely pubescent stems up to 30 cm long, and has shorter, elliptic leaves.

### Phylogenetic analyses

Bayesian inference (BI) and maximum likelihood (ML), yielded highly congruent topologies (Fig. 3). The BI tree is shown with Bayesian posterior probabilities (PP) and ML bootstrap support values (BS) indicated at the nodes.

*Ceropegia gengmaensis* is well supported as belonging to *C.* sect. *Chionopegia* and formed a strongly supported sister relationship with *C. salicifolia* (PP = 1.00, BS = 100). This pair was further grouped with *C. mairei*, indicating a close affinity among these taxa.

Despite this close relationship, *C. gengmaensis* is resolved as an independent lineage with strong statistical support. This clear phylogenetic separation, together with consistent morphological differences, supports the recognition of *C. gengmaensis* as a distinct species.

### Taxonomic treatments

#### Ceropegia gengmaensis

Taxon classificationPlantaeGentianalesApocynaceae

P.R.Luo, A.Liu & H.Sun sp. nov.

28A0DB31-BD8A-5CE1-A4BA-2855AC3707C7

urn:lsid:ipni.org:names:77381438-1

Figs 1, 2, 4

##### Diagnosis.

*Ceropegia gengmaensis* shows affinities with *C. salicifolia* and *C. dolichophylla*, both of *C.* sect. *Chionopegia* . It differs from *C. salicifolia* by its erect to sprawling habit (vs. twining), elliptic leaves 2.0–5.5 cm (vs. lanceolate, 6–15 cm), densely pubescent stems bearing usually solitary flowers (vs. glabrous stems with sessile cymose many-flowered inflorescences), and narrower corolla lobes with only slight expansion above the throat (vs. lobes longer and wider, upper portion markedly expanded). From *C. mairei* it differs by the much less pronounced basal inflation of the tube (vs. a strongly inflated tube) and an annular ring of white hairs at the mouth of the basal inflation (vs. inner surface of the basal inflation with dense white hairs). Compared with *C. dolichophylla*, *C. gengmaensis* is erect to sprawling and shorter (up to 30 cm) with pubescent stems, whereas *C. dolichophylla* is a twining or climbing vine (up to 1.5 m) with glabrous stems and much longer, narrowly lanceolate leaves. They also differ markedly in floral morphology: in *C. dolichophylla* the corolla lobes are pilose inside, often with conspicuous marginal vibratile hairs; whereas in *C. gengmaensis* hairs are mainly restricted to a distinct annular ring at the mouth of the basal inflation of the tube.

##### Type.

**China** • Yunnan Province: Lincang City, Gengma County, 23°36'00"N, 99°05'60"E, 1200 m, 11 May 2025, *P. R. Luo & R. Tong 228* (holotype: KUN1643961).

##### Description.

Perennial, single-stemmed, erect to sprawling herb, up to 300 mm long; occasionally several stems arising separately from a common rootstock, stems rarely branched. ***Roots*** 8–12, white, fleshy, fusiform, fascicled, 80–200 × 2–6 mm. ***Stem*** slender, terete, greenish or brown, 1–2 mm thick, uniformly finely puberulent; internodes 10–40 mm long. ***Leaves*** opposite; petiole terete, 10–15 × ca. 1 mm, finely pilose, adaxially grooved; blade elliptic, 20–55 × 10–25 mm, adaxially green, densely puberulent, abaxially green, pilose along midvein, base cuneate, acute; midvein adaxially flat or slightly depressed; lateral veins in 1–4 pairs, obliquely ascendant, adaxially flat, abaxially slightly convex. ***Flowers*** 1–2, extra-axillary; subsessile; ***pedicel*** 5–10 × 1 mm, finely pilose; bracts 3–5, subulate to lanceolate, 2–4 mm long, greenish, acuminate at apex. ***Sepals*** 5, subulate to lanceolate, 3.0–5.0 × 0.5–1.0 mm, acuminate at apex, glabrous. ***Corolla*** tubular, 30–50 mm long ***tube*** 15–20 × 3–5 mm, outside reddish-purple, glabrous, inside with reddish-purple longitudinal stripes otherwise white except for reddish-purple upper third, white hairs in mouth of basal inflation, otherwise glabrous; basal inflation ovoid, 5–10 × 3–5 mm, gradually narrowed and increasing from the middle to 5–10 mm at mouth, ± 1.5 times as wide as the basal inflation; ***lobes*** 1.0–15.0 × 1.0–3.0 mm, red outside, white inside except for reddish-purple apical third, strongly longitudinally folded outwards along midrib exposing white inner surface with white hairs; ***outer coronal lobes*** ca. 2.0 × 1.0 mm, orange, deeply divided into two triangular, cuspidate teeth, with few white hairs on the inner surface; ***inner coronal lobes*** linear-ligulate, ca. 3.5 × 0.8 mm, light translucent yellow, apices convergent but not fused, slightly recurved. ***Pollinia*** orange, ovoid, ca. 0.25 × 0.30 mm. ***Follicles and seeds*** not seen.

##### Etymology.

*Ceropegia gengmaensis* is named after its type locality, Gengma County, Southwest Yunnan.

##### Vernacular name.

the Chinese name is given as “耿马吊灯花” (gěng mă diào dēng huā) named after the type locality, Gengma County, Southwest Yunnan.

##### Phenology.

Flowering takes place from June to September.

##### Distribution and habitat.

This new species is currently known from Gengma County, Lincang City, Yunnan Province, at altitudes ca. 1200 m, ascending around stones in grassy areas of forest understories. Only one population has been seen, with around 5 individuals.

##### Other specimens examined.

**(all in China)** • *Ceropegia salicifolia*. Yunnan: Ning’er, Afforestation area, limestone thickets, ca. 1620 m, J.D. Ya et al. 14CS9520(KUN1375701!); Yunnan: Xishuangbanna, Mengla, Yiwu, in limestone thickets, elev. ca. 800 m, 8 September 1959, Zhan-Ji Pei 59-9997(KUN0266653! KUN0266654!); Yongde, Wumulong, Ganhe, in limestone thickets, elev. ca. 2350 m, 14 July 2003, En-De Liu 152(KUN0396774! KUN0396776!).

• *Ceropegia dolichophylla*. Yunnan: Baoshan, Shuizhai, Lijiaqing, in slope, elev. ca. 2015 m, 24 August 2021, Wen-Li Zhao & Xiao-Qiang Tao BSGLGSly3103(KUN1595596! KUN1596620!); Yuxi, Xinping, Mopanshan, Jiziqing, Under the forest, elev. ca. 2300 m, 14 June 2017, Li Chen et al. XP456(KUN1573958!).

• *Ceropegia mairei*. Sichuan: Panzhihua, the Cycas National Nature Reserve, Fengjialiangzi, in the thickets of the ridge, elev. ca. 2150 m, 13 August 2016, T. Zhang et al. 16CS12570(KUN1455767); *ibid. loc*. Under the forest, elev. ca. 2182 m, 13 August 2016, T. Zhang et al. 16CS12580(KUN1435889!); *ibid. loc*. Under the forest, elev. ca. 2182 m, 13 August 2016, T. Zhang et al. 16CS12581(KUN1455823! KUN1462749!).

## Supplementary Material

XML Treatment for Ceropegia gengmaensis

## Additional information

### Artificial Intelligence (AI) use

The authors accept full responsibility for the content of the manuscript, including the disclosure of any use of AI.

No AI tools were used in the preparation of this manuscript.

### Author ORCIDs

P-R. Luo https://orcid.org/0000-0001-9621-3366

A. Liu https://orcid.org/0000-0001-6281-7145

R. Tong https://orcid.org/0009-0008-6217-837X

X-J. Zhang https://orcid.org/0000-0002-0668-8497

Z-Y. Wang https://orcid.org/0009-0009-0614-3284

T. Deng https://orcid.org/0000-0003-4163-3368

DJ. Goyder https://orcid.org/0000-0002-3449-7313

H. Sun https://orcid.org/0000-0001-5127-4579

### Data availability

All of the data that support the findings of this study are available in the main text.
